# An Evaluation of Multi-Channel Sensors and Density Estimation Learning for Detecting Fire Blight Disease in Pear Orchards

**DOI:** 10.3390/s24165387

**Published:** 2024-08-21

**Authors:** Matthew Veres, Cole Tarry, Kristy Grigg-McGuffin, Wendy McFadden-Smith, Medhat Moussa

**Affiliations:** 1 School of Engineering, University of Guelph, Guelph, ON N1G 1W2, Canada; 2 OMAFA, 1283 Blueline Road, Simcoe, ON N3Y 4N5, Canada; 3 OMAFA, 4890 Victoria Avenue North, Vineland Station, ON L0R 2E0, Canada

**Keywords:** deep learning, fire blight symptom detection, multi-spectral imaging, pear orchard

## Abstract

Fire blight is an infectious disease found in apple and pear orchards. While managing the disease is critical to maintaining orchard health, identifying symptoms early is a challenging task which requires trained expert personnel. This paper presents an inspection technique that targets individual symptoms via deep learning and density estimation. We evaluate the effects of including multi-spectral sensors in the model’s pipeline. Results show that adding near infrared (NIR) channels can help improve prediction performance and that density estimation can detect possible symptoms when severity is in the mid-high range.

## 1. Introduction

Fire blight is a devastating disease found in apple and pear orchards. If left untreated, it can kill an entire orchard. In Ontario, Canada, fire blight management is a season-long approach with infection risk present from bloom through to harvest in conducive weather. Disease scouting or visual observations of symptoms are common preventative measures enabling early detection and treatment intervention, with some reports recommending, e.g., “Starting at bloom … every 3 to 4 days” for blossom infections [1]. Performing inspections at this recommended frequency is challenging due to the large scale of many orchards and the need for trained scouts to perform the inspections. As such, developing an automated scouting system for fire blight detection could have a positive impact on the current growing operations.

One of the most characteristic signs of fire blight is a scorched-like patterning which can often be seen on the bark and leaves of a tree. These symptoms, however, typically represent a later stage of infection, and the challenge is to identify “strikes” for sanitation practices (pruning) as early as possible so that growers can treat it. Several studies have been proposed which have laid the groundwork for detecting these symptoms using alternative imaging strategies, such as multi- or hyper-spectral means, including leaves of pear trees by [2] and apple trees by [3]. The use of multi-spectral imaging in our work is motivated by trends of lower reflectance values around the 710–850 nm range for infected leaves, as shown by [3]. Other works, such as [4], have also shown the application of hyper-spectral imaging for classifying apple valsa cankers. The latter, however, is typically carried out before leaves form and is outside the scope of this paper.

In this paper, we approach the scouting operation as a micro-scale detection problem where the focus is on detecting individual possible symptoms within a tree rather than infected trees as a whole. In addition, we are interested in investigating the use of multi-spectral modalities within a deep learning pipeline while targeting symptom detection. As such, we developed a custom data collection rig equipped with RGB (400–670 nm) and near infrared (NIR) (700–800 nm, 820–1000 nm) sensors, and using real-time kinematic (RTK) GPS locations to geo-tag all images. A density estimation approach using deep learning was used to detect symptoms of fire blight directly from collected side-view images of orchard trees. Images used in this work were collected from a pear orchard in the Niagara region of Ontario, Canada, over four separate dates, June–August 2022.

### Contributions

1. We propose a density estimation approach for learning to recognize possible symptoms of fire blight. As a learning approach, this formulation reduces the need for precise and time-consuming bounding box annotations from traditional object detection approaches which can help facilitate the construction of larger dataset sizes.
2. In contrast to canopy-based approaches for scouting, we focus on the use of ground-level imagery for detection. This approach allows for targeting individual symptoms which may not be visible to cameras used in aerial image-based systems.
3. Alternative imaging modalities such as NIR have shown promise for being able to detect fire blight symptoms in leaves [2,3]. In this work, we investigate the use of multiple-image modalities to enhance detection results at different points of the spring/summer growing season.

## 2. Background and Literature Review

### 2.1. Fire Blight

Fire blight is caused by the bacterium *Erwinia amylovora*, and hosts of the disease can include apple, pear, hawthorn, crabapple and Japanese or flowering quince, mountain ash, cotoneaster, raspberry, serviceberry, and spirea. The bacteria that cause fire blight overwinter at the edges of cankers on trunks and limbs. In the spring, bacteria in cankers become active, and droplets containing high numbers of bacteria ooze out of the infected bark tissue. The ooze can be rain-splashed or carried by wind or insects to open blossoms and tender developing shoot tips. Secondary infection occurs throughout the growing season and is caused by the spread of the bacteria from infected tissue to newly developing shoots or wounds made by insects, wind, hail, or spread from contaminated tools. When favourable environmental conditions occur, the bacteria pass through natural openings, including open blossoms, tears, or cuts in the foliage and/or woody tissue, or insect damage directly into the host tissue.

Symptoms are often referred to by the part of the tree affected, such as blossom, shoot, fruit, or rootstock blight. In general, infected tissue of blossoms and shoots wilt, shrivel, and turn brown or black, giving an appearance of being scorched by fire. Once infection occurs, the disease moves quickly through the vascular tissue into other succulent tissues (one- to two-year-old wood), especially if accompanied by warm, humid conditions. The most severe losses from both blossom and shoot blight occur when the disease progresses into older wood, forming cankers which can girdle the branch or trunk and cut off transport of nutrients and water. Without preventative measures, the pathogen can move through highly vigorous trees from infected blossoms to the roots in one month under favourable weather conditions (20–28 °C). Removal of infected tissue from the orchard is the best management practice for controlling fire blight. Pruning symptoms as they develop and during dormancy helps to reduce the inoculum load within the orchard, reducing the impact of the disease in the growing season and improving the efficacy of pest control products.

### 2.2. Orchard and Orchard-Scale Scouting Efforts

Studies in recent years have explored RGB and multi-spectral/hyper-spectral imagery not just for individual sample identification, as above, but also in the context of scouting within the larger orchard environment. These approaches often leverage aerial imagery and train classifiers to distinguish between healthy/infected samples, possibly through features extracted via vegetation indices [5,6,7,8,9]. In [10], Kang, Kim, and Noh show how representation learning frameworks via deep learning can be used to predict spatial output maps (the same resolution as the aerial images) where symptoms are likely to be present. While aerial imaging and detection in this way can efficiently cover the full orchard, it is still unclear how well the practice will apply to early-stage detection when symptoms are few in number and may not be visible to the camera.

Classification of severity is also an open problem, as in [6], where the authors represented severity as the ratio of infected and healthy pixels for a tree. In this way, carefully identifying the boundaries of each tree is also an important step in the model pipeline. Side-view images of trees and manually extracted features have also been investigated with respect to disease severity [5]. In that work, Jarolmasjed et al. presented an experiment where features of “maximum length of shoots with senesced leaves (pixels), the total area of senesced leaves (pixels), and the ratio of senesced to healthy leaf area” were found to have some correlations with disease severity. In our current work, we seek an approach that is scalable to the full orchard and in which symptoms could be more directly observable through side-view images.

### 2.3. Deep Learning for Object Detection

In this work, we seek an approach which can target individual symptom detection directly. With respect to learning-based approaches, object detection models including the Single-shot Detector (SSD) [11] or the YOLO [12] family of models have already seen extensive use in problems such as disease detection on tree leaves [13] or fruit detection [14,15] for purposes such as yield estimation, and they could be seen as a natural approach to fire blight detection.

A recent investigation by Mahmud et al. [9] trained a model known as Mask R-CNN [16] to detect symptom “spots” of fire blight in apple orchards. This model predicts both a bounding box around symptoms, as well as an object mask for which pixels within the box belong to the target object. Similar to our current investigation, the approach used side-view images of trees to output predictions; however, the model was only trained using RGB images and with images captured by portable cameras.

From an implementation perspective, however, there are a number of known limitations with respect to labelling effort with these approaches. Pure object detection requires bounding boxes (two clicks per symptom forming a tight box around the target), or in the case of instance segmentation, polygon-based annotations (tens to hundreds of mouse clicks), which create a tight pixel-wise mask around the target object. In our work, we seek an approach which scales well with the number of symptoms which may be encountered during severe stages of infection.

### 2.4. Deep Learning for Density Estimation

In the task of disease scouting in orchards, some constraints on exact localizations within the image can be relaxed if we assume that any positively identified areas of infection will be manually examined by the grower for treatment. As long as the grower is guided to the approximate location, scouting can be formulated as a density estimation task [17] where machine learning (e.g., deep learning) is in effect used to count the number of objects in an image. Density estimation uses a labelling strategy which is much more amenable to the quantity of symptoms which may be encountered (requiring a single pixel label per instance). In contrast to the polygon representations used in [9] for symptoms, the density estimation formulation allows us to label every individual symptom with only a fixed increase in complexity.

In modern applications, deep learning models are typically trained to predict the spatial density of objects by learning image-to-image mapping e.g., [18]. In broader agricultural applications, density estimation has been adopted to help count the number of flowering pineapple plants [19], to detect both citrus [20] and eucalyptus trees [21], to estimate the density of cattle in images [22], and to count the number of pests on images of leaves [23]. Our work seeks to investigate the suitability of this approach for disease detection throughout the growing season for pear trees.

## 3. Data Collection

A pear orchard located in Niagara, Ontario, Canada, was imaged on four separate dates in the spring/summer of 2022. We refer to these dates using YYYY-MM-DD format, and name them 2022-06-02, 2022-06-22, 2022-07-13 and 2022-08-09 respectively. The start of these dates corresponds to the period of time beginning after leaves have appeared on the trees. We focus on recognizing possible symptoms of the disease through a visual inspection of pear leaves.

### 3.1. Data Collection Methodology

**Figure 1:**
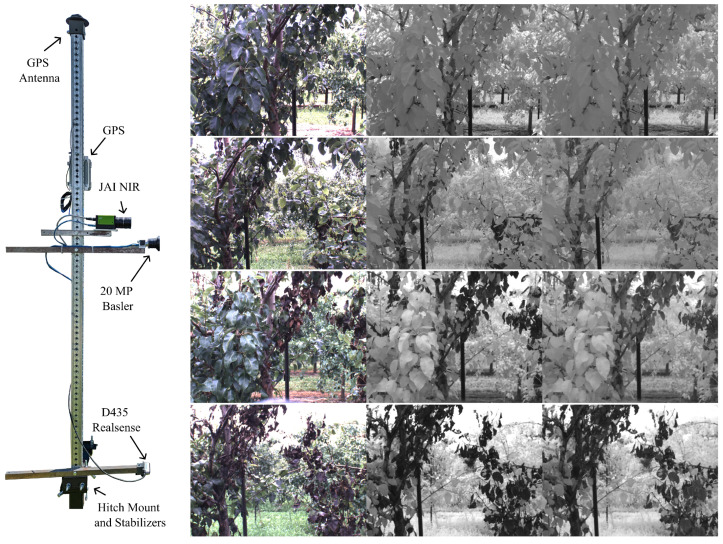
Data collection apparatus and sample images from the JAI camera taken at the same location in the orchard at different parts of the season. **Top to bottom**: 2022-06-02, 2022-06-22, 2022-07-13, and 2022-08-09. The columns correspond to RGB, NIR1, and NIR2 channels, respectively.

A machine vision system was developed to facilitate ground-based imaging of the full orchard. The system was designed to be mounted on the back of a pickup truck, record data from multiple sensors simultaneously, and store all information in a database. The system itself is composed of a vertical mast having an RTK-GPS and three separate cameras mounted at varying heights, including a 20 MP RGB camera (capturing a large image of the tree at a high resolution), a RealSense camera (capturing an RGB-D image of the base of the tree), and a JAI RGB-NIR (JAI) camera capturing three registered images of the scene using RGB (400–670 nm), NIR1 (700–800 nm), and NIR2 (820–1000 nm) wavelengths. The JAI camera was installed at eye level, approximately 5.5 ft above the ground. In this work, we focus on images from the JAI camera only due to their pixel-wise alignment across sensor channels. This alignment allows us to label a single image and have the labels propagated through to the remaining two sensors and ensures that each sensor captures the exact same scene. Figure 1 shows an example of the data collection system and the sample images from the JAI camera.

**Figure 2:**
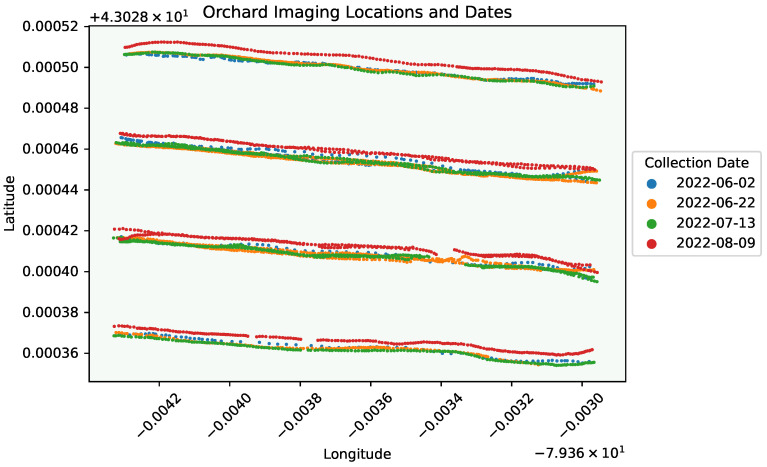
Orchard rows where images (dots) were taken. Slight path offsets are visible between each date due to minor pathing differences, including differences due to obstacles which may have been present. Both forward and reverse paths were followed for the inner rows. Best viewed in colour.

Figure 2 shows the locations in the orchard where images were collected across each of the four dates. During data collection, every tree in the orchard was imaged regardless of whether it had visible symptoms of fire blight or not. Multiple images of the same tree at slightly different locations were collected. Images were collected at a resolution of 1536×2048 pixels (h×w). Images were collected under varying conditions; some days were sunny, while others were overcast. The spacing of the trees, the imaging direction, and the density of the canopy are all different sources of variation.

### 3.2. Field Ground Truthing and Target Features

Trees exhibiting symptoms of fire blight were monitored by expert scouts over the growing season, and approximate areas of infection were labelled with flagging tape tied around the trees’ limbs. Due to the scale of the orchard, only certain locations within the orchard were routinely scouted. Using these ground truth labels as a reference, all collected data were labelled by non-expert personnel in an offline environment.

**Figure 3:**
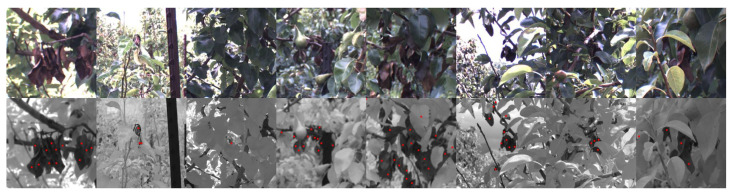
Sample symptoms which were labelled in the dataset. Images are crops of their larger counterparts. **Top row**: RGB image. **Bottom row**: NIR1 image. The red dots in the NIR1 images represent labelled instances’ ([x,y] locations).

Figure 3 shows a set of image crops randomly taken from around labelled instances in our dataset and illustrates the types of visual features which were targeted. The red dots indicate individual symptom-like objects in the image which have been labelled. In general, in the RGB images, these included groups of red-tinged leaves, and in the NIR images, they included leaves or fruit which had a noticeably lower reflectance value than their surroundings. We note that our current work is a modelling approach for wide-scale scouting and symptom detection; we do not perform any destructive testing to verify the presence or absence of the disease. During labelling, we erred on the side of caution and labelled any instance that could be a potential symptom. Labelling was performed using Label Studio [24], and both the RGB and NIR1 images were available for reference.

### 3.3. Dataset Construction

**Figure 4:**
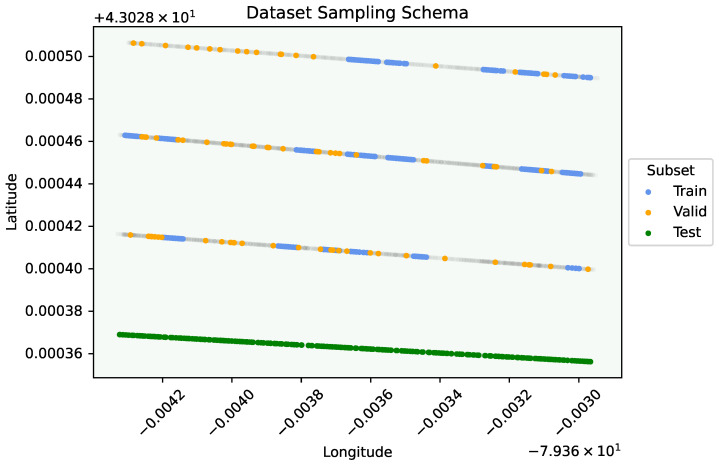
All orchard imaging locations (grey), and training, validation, testing subset allocation.

Figure 4 shows the scope of the orchard scanning process, as well as how the dataset was chosen and labelled for training, validation, and testing splits. To carry this out, we applied the following methodology:

- **Training Set** During a separate investigation in March earlier that year, the same orchard was imaged and ground-truthed by scouting experts. We used the location of possible early symptomatic locations to focus our current investigation and labelled images from the spring/summer season falling within 5 m of these locations.
- **Validation Set** Using the portion of data that was not sampled for training, we created a date-balanced validation dataset by sampling 20 images from each collection date.
- **Testing Set** Using the GPS data, images from a single tree row (and corresponding imaging direction) in the orchard were selected from the data as an independent test set. For simplicity, every second consecutive image was labelled.

Figure 4 shows an example of how images were sampled for the different training, validation, and testing subsets. As we describe later in Section 4.2, we will require knowing the location in the orchard where each sample was taken. To carry this out, we calculated a single vector for each orchard row (grey) and projected each sampling point to its nearest location along this line, which allowed us to query locations in the row by using its distance from the row entrance. The final dataset size and label statistics can be seen in Table 1. Note that in the training set, all images are required to have at least one labelled instance. In the validation and testing sets, we do not enforce this constraint and we seek to observe the model’s performance as if it was performing detection in the field where symptoms may or may not be present in any given image. The relatively smaller number of labels/image for the testing set is likely due to this sampling strategy, as well as being away from the influence of the rest of the orchard.

## 4. System Architecture and Methodology

**Figure 5:**
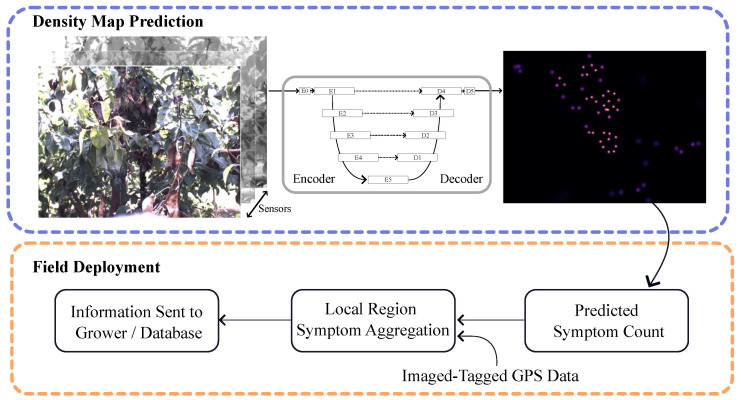
Overall model pipeline. A U-Net model receives an input image having n−channels (**left**) and produces an output density map (**right**). The density map can be summarized to obtain a prediction of the number of symptoms, which can be aggregated using GPS data to monitor the orchard’s health. A brighter intensity in the density map represents larger values.

To perform orchard-wide health monitoring, we broke our approach down into two phases: density map prediction and field deployment. The density map prediction was handled by a U-Net [25]-based deep learning model, while the field deployment used image-tagged metadata (GPS locations) to aggregate predictions and propose symptomatic areas of the orchard which require manual inspection by the grower. Figure 5 shows an illustration of our approach.

### 4.1. Density Map Prediction

The U-Net architecture was originally proposed for the purpose of biomedical image semantic segmentation [25]. It consists of encoder and decoder structures, which define how the data flows throughout the model. The encoder module is used to process and downsample images to a highly compressed representation ($E_{5}$, Figure 5); the decoder, on the other hand, takes this compressed representation, along with skip connections from the encoder, and attempts to produce (i.e., decode it) an output image having the same spatial resolution as the input. In our implementation, the output image is represented as a single-channel, continuous 2D image showing the spatial density of symptoms in an image. In Figure 5, the brighter the value on the output map, the higher the density of symptoms. We refer the reader to [25] for further information on the U-Net architecture.

The inputs to the model can be any n−channel 2D image. In our work, we experiment with different input compositions: we consider 2D, n−channel images composed of RGB, NIR1, NIR2 sensors (or combinations of such) captured from the JAI camera.

#### Density Map Creation

To create the target values for the density map, recall from Section 3.2 that our labels are represented by (x,y) pixel coordinates denoting possible symptoms. Given a list of these coordinates for an image, we first create a binary output image (with the same spatial resolution as the input), where each pixel is toggled between (0, 1), depending on whether a symptom was recorded at that location or not. This spatial mask is then smoothed using a 2D Gaussian filter to yield continuous target values. The goal for the network is to predict these spatial continuous values.

### 4.2. Deployment and Post-Processing

Given the predicted density map from the model, the map can be summed to obtain a prediction on the number of symptoms present in the image. Any one image of a tree could in theory be used to signal to the grower whether symptoms are present. However, depending on factors such as the image sampling rate (potentially capturing multiple images per tree or at different angles) along with the uneven spacing of trees, this approach could provide a false signal. Instead, we leverage the GPS information tagged on every image to bin predictions into fixed-sized buckets. As the trees are planted in fixed rows, we first identify a location in each row to serve as the “row entry”, and then we calculate a distance from this location to every other image taken from within the same row. We then create fixed-sized bins (e.g., every 2 m), assign every image to the corresponding bin, and then aggregate all predictions for each bucket.

## 5. Experiments

### 5.1. Implementation Details

All models are built using the Segmentation Models Pytorch (SMP) library [26]. Due to the size of our dataset, we use transfer learning and fine-tune weights for a ResNet50 encoder model, which was pre-trained on the ImageNet dataset. This model is used extensively in the literature, and since our focus in this paper is not on comparing models, we decided to use only this model. This allowed us to focus on sensor input representations and their impact on the model’s output. Using different models may lead to different performance and will be investigated in our future work.

To prepare the target density map images, we used the code provided by [27]. We used the mean-squared error (MSE) loss for learning, a learning rate of 1 × 10^−4^, and trained for 20 epochs using the Adam optimizer. All images were downscaled from their original resolution by a factor 4× to a size of 384×512 pixels, allowing us to train over a minibatch of examples, which we empirically set to 12. We also performed data augmentation in the form of horizontal image flipping (*p* = 0.5) during training and used the validation set (which was balanced across data collection dates) to determine where to stop training.

### 5.2. Experiment 1: The Effects of Input Modalities on Detection Accuracy

The JAI camera collects images from RGB, NIR1, and NIR2 sensors. To understand the effects of different sensors on the prediction accuracy, we test the following four combinations of sensors: RGB, NIR1, RGB + NIR1, and RGB + NIR1 + NIR2. For every sensor combination, we repeat the training procedure 10 times using different seeds and report the average results. We use the mean absolute error (MAE ) metric for reporting our model’s performance:

(1) $$\mathrm{MAE}=\frac{1}{N}\sum_{i=1}^{N}\left|y_{i}-{\bar{y}}_{i}\right|$$

where ${\bar{y}}_{i}$ represents the predicted count and $y_{i}$ represents the ground truth values. Given that symptom counts and severity may change throughout the season, for each experiment, we break the analysis down by date that the data were collected.

**Figure 6:**
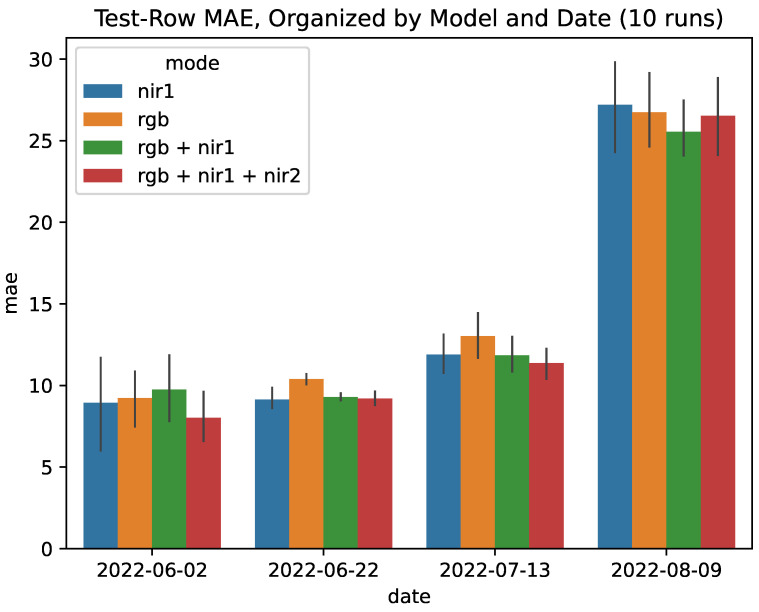
Mean absolute error of models trained with different sensor channel combinations. Results are reported across multiple runs with different seeds. We consider the RGB-trained model to be the baseline.

Figure 6 shows our results. Note the MAE for models on both the earliest (2022-06-02) and latest (2022-08-09) dates, which reflect the extremes of the dataset in terms of symptom visibility and quantity (c.f., Table 1). While we expect images with many symptoms to be challenging, there is a comparatively large MAE on the earliest date as well: the MAE is reported to be around 10 for all models, which could be a sign of over-prediction. During the middle two dates, predictions averaged from each of the separate runs are fairly similar. The RGB model can be seen to have the worst MAE during these dates as well, but only by a small margin and generally within the overall error range. It can also be seen that training the model on different sensor combinations had a slight effect on the model’s performance. Across all tested sensor combinations, adding either NIR1 or NIR1 + NIR2 bands to the RGB channels improved the model’s MAE. These results are promising, but more work is needed to ensure robust detection under various conditions.

**Figure 7:**
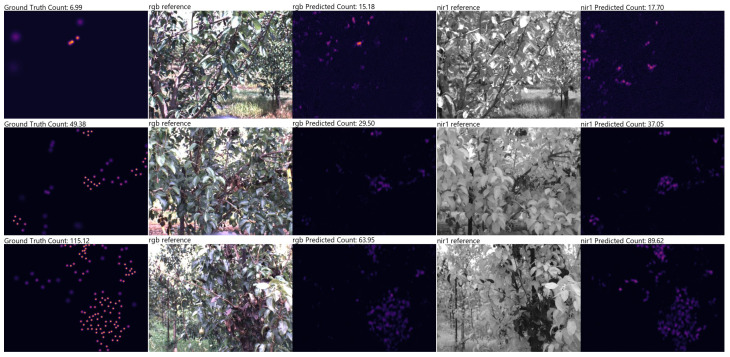
Sample results of RGB and NIR1 models trained on the same seed. The ground truth map can be seen in the left column. Sub-figures were normalized independently for visibility.

Sample predictions from the RGB and NIR1 models can be seen in Figure 7. In these images, the left-most column represents the ground truth density map, while the remaining two columns (from left to right) show the RGB input image and predicted density map, as well as the NIR1 input image and predicted density map, respectively. In the first row, the model tends to be a little over-eager in the predictions. In the second and third rows, the models generally seem to make strong predictions where larger clusters of blight symptoms can be seen, but the overall symptom count is less than what is in the ground truth.

### 5.3. Experiment 2: Localizing Predictions within the Orchard

**Figure 8:**
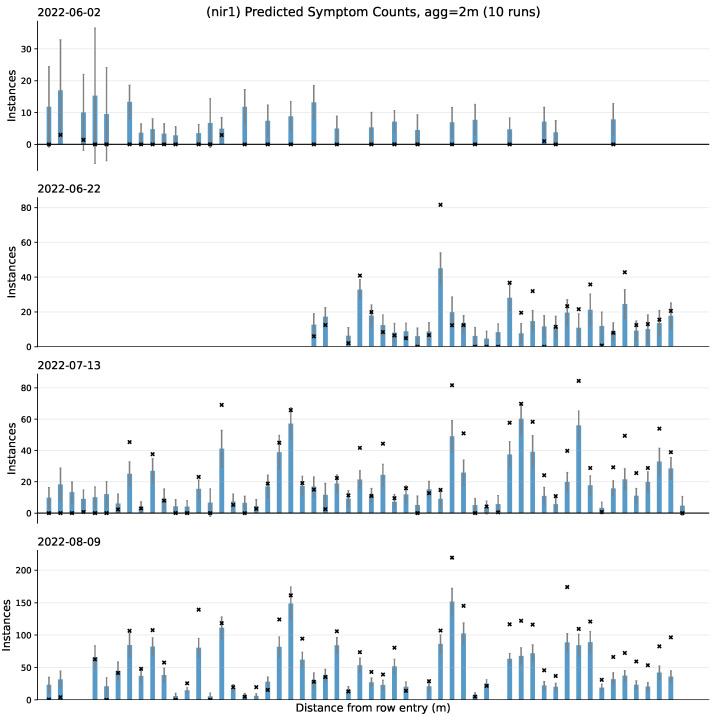
Visualizing ground truth and predicted symptom counts for an entire orchard row. Each bar corresponds to a 2 m binned area of the orchard. x’s denote the ground truth symptom counts. Missing data from the first half of 2022-06-22 are attributed to an error which prevented images from being saved properly during data collection. Note the different scales used on the y-axis across the various dates.

Our model makes predictions of possible symptoms using the side-view images of trees. To convert these predictions to a health report for the whole orchard, we follow the procedure in Section 4.2 and both bin and aggregate predictions using their relative locations from within an orchard row. Figure 8 shows the results of aggregation for the NIR1-trained model, where the ground truth symptom counts are marked as a black × and predicted counts are shown as blue bars. Note how for the earliest date (2022-06-22), most of the predictions are greater than the ground truth value. The latter dates on the other hand show values which may be either over or under. By continuously monitoring predictions across time, we can visualize how trends in symptoms change. In this Figure, this trend can be observed by reading the predictions vertically.

There are also other insights that can be seen from this Figure. For example, there is a slight sparsity in bins for the date 2022-06-02, which can be attributed to the image capturing frequency (c.f., Figure 2) and becoming familiarized with the data collection procedure. For a system which makes predictions throughout an orchard, it is critical to ensure every location is scanned properly; incorporating, e.g., an encoder (which takes images at fixed distance intervals) could be a possible solution for mitigating this sparsity.

Lastly, with respect to the date 2022-06-02, there is a gap that can be seen between the number of labels and the number of predicted symptoms. From Table 1, images on this date already have very few objects which could resemble plausible symptoms. This, combined with an already small subset of data could make recognition difficult and could also be a contributing factor for the high prediction variance.

### 5.4. Experiment 3: Converting Predictions into Binary Classifications

Given predicted symptom counts, these estimates can be further refined to provide a signal to the grower of whether regions of the orchard need to be inspected or not. From the ground truth values, we can convert count values into a boolean (inspect, no-inspect) signal by assuming that any image with at least one labelled value represents that symptoms are present. Then, from the predicted values, we can find thresholds which minimize the false-positive rate (FPR) and the false-negative rate (FNR):

(2) $$\mathrm{FPR}=\frac{\mathrm{FP}}{\mathrm{FP}\;+\;\mathrm{TN}}\;\mathrm{FNR}=\frac{\mathrm{FN}}{\mathrm{FN}\;+\;\mathrm{TP}}$$

where FN, TP, FP, and TN represent the standard false-negative, true-positive, false-positive, and true-negative values in a binary classification.

**Figure 9:**
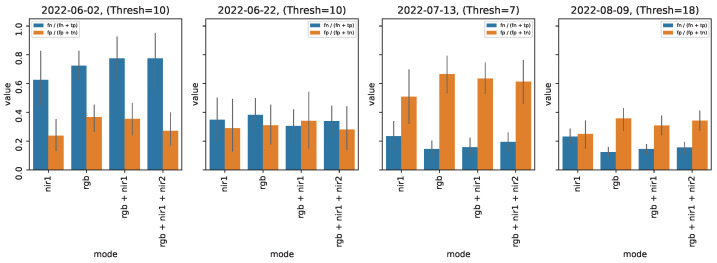
Measuring model performance in terms of wrong predictions.

Intuitively, FPR looks at how often fire blight is predicted when there is none, and FNR looks at how often no fire blight is predicted when it is present. Figure 9 shows the FPR and FNR across each date and trained model, averaged over the 10 runs. A high FNR (blue bars) on the earliest date can be seen, which indicates that if symptoms are present in the image, the model may fail to catch it. Similar to Experiment 2, if we recall from Table 1 that this date had a relatively small number of labels per image compared to the rest of the dataset, this result is not entirely surprising. It does however help evaluate the limits of our current system which fairs much better across the remaining data collection days. We discuss methods which may be helpful in improving the model’s detection performance in Section 6.

## 6. Discussion and Conclusions

Figure 7 shows an example of predictions made by our model. In places with a high density of possible symptoms, our model can be seen to generally predict values in nearby regions at similar intensities. By focusing on symptom localization within an orchard (and not within a specific tree), there is an acceptable tolerance with respect to precise localization within an image, and which enables the applicability of a point-wise labelling approach and learning via density estimation, as explored here. Compared to approaches which focus on detection from aerial images, our approach allows for learning visual features of individual symptoms and possible detection during earlier stages as we directly inspect each tree from within the row. There are also benefits with respect to approaches which leverage classical models such as support vector machines or decision trees and which may require special preprocessing of the input data and manual feature engineering. Our current approach is designed to benefit from both: efficient wide-scale imaging with minimal manual supervision.

Experiment 1 showed how the density estimation models performed when given images with both high and low numbers of possible symptoms. Our findings suggest that it may be difficult to perform a density estimation when modelling in the extreme low-count regime (2022-06-02) when only a few instances are present. In the high-count regime (2022-08-09), there were noticeably higher MAE values; part of this could be attributed to the increase in symptom counts between the various dates (c.f., Table 1, an increase from 23.16 to 66.13 labels/image for the last two dates) and the model working to learn across all different configurations.

We did notice occasional instances where trees in the background could have symptoms predicted as well, and which could have contributed to some of the variations seen in Experiment 3. To further improve accuracy, some form of background removal could potentially be beneficial, whether trained through deep learning or via other means, such as depth thresholding. There is also a potential to employ temporal or multi-temporal models to enhance accuracy as suggested by [6]. However, we will leave this for a future study.

In Experiment 3, we presented a method for converting predicted count values to a signal which can be directly used by the growers. However, the results show the difficulty in finding a suitable threshold for the earliest collection date. Improving early detection is a key issue for future development efforts; in terms of value to the growers, the proposed system can be most effective if it captures changes in the leaves before they turn dark brown, which, by that time, can be easily seen by the growers. This is challenging since it is difficult to label instances at a very early stage manually. In our work, a temporal approach could be used to work backwards from signs of heavy infection towards earlier points in the season. This in turn could help enable automated labelling for regions with confirmed symptoms at later dates. One challenge here is ensuring exact alignment between images at various dates. While we were able to take images from similar locations in the orchard across different dates, images were not always taken at the exact same position and height. Other strategies which focus solely on the earliest possible detection could try using bounding box detection methods, where the number of symptoms is small and bounding boxes may be viable. As part of our future work, we will also seek to evaluate different model architectures and weight configurations to further optimize detection performance.

## Figures and Tables

**Table 1:** Composition of training, validation, and testing datasets.

	Training		Validation		Testing	
**Date**	**Images**	**Labels/Image**	**Images**	**Labels/Image**	**Images**	**Labels/Image**
2022-06-02	22	2.72	20	2.35	28	0.36
2022-06-22	159	32.42	20	33.45	43	16.30
2022-07-13	135	44.81	20	48.75	87	23.16
2022-08-09	29	94.69	20	86.05	87	66.13
Total	345		80		245	

## Data Availability

Restrictions apply to the availability of data used in this paper.
